# ZEB1 Mediates Acquired Resistance to the Epidermal Growth Factor Receptor-Tyrosine Kinase Inhibitors in Non-Small Cell Lung Cancer

**DOI:** 10.1371/journal.pone.0147344

**Published:** 2016-01-20

**Authors:** Takeshi Yoshida, Lanxi Song, Yun Bai, Fumi Kinose, Jiannong Li, Kim C. Ohaegbulam, Teresita Muñoz-Antonia, Xiaotao Qu, Steven Eschrich, Hidetaka Uramoto, Fumihiro Tanaka, Patrick Nasarre, Robert M. Gemmill, Joëlle Roche, Harry A. Drabkin, Eric B. Haura

**Affiliations:** 1 Department of Thoracic Oncology, H. Lee Moffitt Cancer Center and Research Institute, Tampa, Florida, United States of America; 2 Department of Molecular Oncology, H. Lee Moffitt Cancer Center and Research Institute, Tampa, Florida, United States of America; 3 Department of Biomedical Informatics, H. Lee Moffitt Cancer Center and Research Institute, Tampa, Florida, United States of America; 4 Second Department of Surgery, School of Medicine, University of Occupational and Environmental Health, Kitakyushu, Fukuoka, Japan; 5 Division of Hematology-Oncology, Department of Medicine and the Hollings Cancer Center, Medical University of South Carolina, Charleston, South Carolina, United States of America; University of Parma, ITALY

## Abstract

Epithelial-mesenchymal transition (EMT) is one mechanism of acquired resistance to inhibitors of the epidermal growth factor receptor-tyrosine kinases (EGFR-TKIs) in non-small cell lung cancer (NSCLC). The precise mechanisms of EMT-related acquired resistance to EGFR-TKIs in NSCLC remain unclear. We generated erlotinib-resistant HCC4006 cells (HCC4006ER) by chronic exposure of *EGFR*-mutant HCC4006 cells to increasing concentrations of erlotinib. HCC4006ER cells acquired an EMT phenotype and activation of the TGF-β/SMAD pathway, while lacking both T790M secondary *EGFR* mutation and *MET* gene amplification. We employed gene expression microarrays in HCC4006 and HCC4006ER cells to better understand the mechanism of acquired EGFR-TKI resistance with EMT. At the mRNA level, *ZEB1 (TCF8)*, a known regulator of EMT, was >20-fold higher in HCC4006ER cells than in HCC4006 cells, and increased ZEB1 protein level was also detected. Furthermore, numerous *ZEB1* responsive genes, such as *CDH1 (E-cadherin)*, *ST14*, and *vimentin*, were coordinately regulated along with increased *ZEB1* in HCC4006ER cells. We also identified ZEB1 overexpression and an EMT phenotype in several NSCLC cells and human NSCLC samples with acquired EGFR-TKI resistance. Short-interfering RNA against *ZEB1* reversed the EMT phenotype and, importantly, restored erlotinib sensitivity in HCC4006ER cells. The level of micro-RNA-200c, which can negatively regulate ZEB1, was significantly reduced in HCC4006ER cells. Our results suggest that increased *ZEB1* can drive EMT-related acquired resistance to EGFR-TKIs in NSCLC. Attempts should be made to explore targeting *ZEB1* to resensitize TKI-resistant tumors.

## Introduction

Despite the benefit of epidermal growth factor receptor-tyrosine kinase inhibitors (EGFR-TKIs) in non-small cell lung cancer (NSCLC) patients with *EGFR* mutation [1], acquired resistance to these therapies is a critical clinical problem. Although the T790M secondary *EGFR* mutation [2] and *MET* gene amplification [3] may together account for 70% of this resistance, mechanisms for the remaining 30% are unclear. The epithelial-mesenchymal transition (EMT) has been negatively associated with EGFR-TKI sensitivity in NSCLC [4–7]. In line with these results, recent studies reported EMT as a possible mechanism of acquired EGFR-TKI resistance in NSCLC cell line models [8,9]. Furthermore, EMT was observed in a subset of NSCLC patients who developed EGFR-TKI resistance [10,11]. However, detailed mechanisms of EMT-related acquired resistance to EGFR-TKIs in NSCLC, as well as the strategies for overcoming it, remain unclear [8,9]. Several signaling pathways, such as FGFR [6,12], TGF-β [8,9], and WNT [13], as well as transcription factors, such as the Zinc finger E-box-binding homeobox 1 (ZEB1) [14], have been implicated in the EMT process.

The EMT enables epithelial cells to gain a mesenchymal phenotype associated with increased migration (for reviews [15–20]). It is an essential mechanism for plasticity during development and tissue repair. It is involved in wound healing, fibrosis, and stem cell biology and contributes to the progression of diseases-like organ fibrosis and cancer. EMT is activated in cancer cells and involved in invasion, metastasis, stem-like properties, and resistance to conventional antineoplastic therapies [15,21,22]. EMT is induced by TGFβ, other growth factors, and hypoxia and involves transcription factors like Snail, Twist, ZEB1/ZEB2, and E12/E47 to modify the transcriptional machinery, alteration of translation and protein stability, expression of non-coding RNAs, and alternative splicing [16,23,24]. Classical features of EMT are loss of cell-cell adhesion and cytoskeletal reprogramming. Low E-cadherin and high vimentin and N-cadherin expressions are classical EMT markers. On the E-cadherin promoter, the histone demethylase LSD1 associates with Snail, the transcription factor involved in early steps of EMT induction, suggesting epigenetic modifications during EMT [25,26]. Indeed, H3K27 acetylation was decreased in ZEB1-induced EMT in lung cancer cells [27]. Recently, molecular features associated with EMT were defined by an integrative approach in lung adenocarcinoma and pointed to an association between cytoskeletal and actin-binding proteins, the EMT phenotype and invasive properties [28]. Interestingly, EMT is transient and reversible, and novel clinical therapeutics targeting EMT are under development [29].

We established HCC4006ER (erlotinib-resistant) cells as a model of EMT-related acquired resistance to EGFR-TKIs by chronic exposure of sensitive HCC4006 NSCLC cells containing an *EGFR* mutation (exon 19; L747-A750del insP) to increasing concentrations of erlotinib. We examined global changes in gene expression to identify molecules and pathways that might contribute to EMT-related acquired EGFR-TKI resistance in NSCLC. In addition, the expression level of micro-RNA-200c (miR-200c) was examined based on reports that miR-200c negatively regulates ZEB1 and the EMT process [30–32].

## Materials and Methods

### Reagents

LBH589, erlotinib, BIBW2992, WZ4002, BEZ235, and AZD6244 were purchased from Chemie Tek (Indianapolis, IN). PD173074, LY364947, salinomycin, and IWP2 were purchased from Sigma-Aldrich (St. Louis, MO). CNTO328 was provided by Centocor, Inc. (Horsham, PA). CL-387,785 was purchased from AXXORA (San Diego, CA). Stock solutions of these reagents in 100% DMSO were diluted directly into the media to indicated concentrations. Human TGF-β1 was purchased from R&D Systems (Minneapolis, MN).

### Cell culture

HCC4006, H1975 and H358 cells were obtained from ATCC (American Type Culture Collection). Cell identity was verified by STR analysis (ACGT, Inc., Wheeling, IL), and the cells were confirmed to be mycoplasma negative by PlasmoTest Mycoplasma Detection (InvivoGen, San Diego, CA). Cells were maintained in RPMI 1640 medium supplemented with 10% fetal bovine serum (FBS; Invitrogen, Carlsbad, CA) at 37°C and 5% CO_2_.

### Generation of EGFR-TKI-resistant cells

HCC4006ER (erlotinib-resistant) cells were generated by exposure of HCC4006 cells containing an *EGFR* mutation (exon 19; L747-A750del insP) to gradually increasing concentrations of erlotinib, beginning at 3 nM, for 3 months. After initial adaptation, the erlotinib concentration was gradually increased to 4 μM. H1975 BIBW-R and H1975 WZ-R cells were generated by exposure of H1975 cells containing an *EGFR* mutation (exon 21; L858R and exon 20; T790M) to gradually increasing concentrations of BIBW2992 (irreversible EGFR-TKI afatinib) or WZ4002 (T790M selective EGFR-TKI), beginning at 3 nM, for 3 months. After initial adaptation, the BIBW2992 or WZ4002 concentration was gradually increased to 3 μM or 15 μM, respectively. Single cell clones of these cells were obtained by seeding at very low density.

### Status of *EGFR* and *KRAS* mutations

Total genomic DNA from parental and resistant cells was prepared using the DNeasy Blood & Tissue Kit (Qiagen, Valencia, CA) in accordance with the product manual. Direct DNA sequencing was used to detect *EGFR* and *KRAS* mutations as previously described [33]. We also applied the PCR-invader assay to look for minor populations of T790M mutant cells, as previously described [34].

### Cell viability assay

Cell viability was determined using the CellTiter-Glo^®^ Luminescent Cell Viability Assay (Promega, Madison, WI) in accordance with the manufacturer’s recommendations. Briefly, cells were plated at 3x10^3^ cells per well in black wall 96-well plates (NUNC, catalog no. 165305; Rochester, NY) and incubated overnight in RPMI with 5% FBS. Cells were then exposed to serial dilutions of inhibitors for 72 hours. Fifty microliters of Cell-Titer Glo Reagent were added to each well, and luminescence was recorded using a VICTOR plate reader (PerkinElmer, Waltham, MA). Results were converted to percent cell viability by comparing treated with untreated (100% viable) cultures from three independent experiments performed in triplicate. Combination Index (CI) at IC50 dose of combination treatment was calculated by CompuSyn software (http://www.combosyn.com/; ComboSyn, Paramus, NJ). CI>1, CI = 1, and CI<1 indicate antagonistic, additive and synergistic effects, respectively [35].

### RTK array

HCC4006 and HCC4006ER cells were incubated in RPMI medium with 5% FBS for 24 hours until ~80–90% of cell confluence. Phosphorylation levels of multiple receptor tyrosine kinases (RTKs), including those of the EGF, FGF, PDGF, insulin, VEGF, EPH, AXL, and MER families (among others), were examined using the Proteome Profiler Human Phospho-RTK array kit (R&D Systems) as previously described [3].

### Protein expression analysis

Cells were grown to ~80–90% confluence prior to harvest and lysis. Western blot analysis on whole cell lysates was performed as described previously [36]. Nuclear extracts for detection of transcription factors were prepared as previously described [37]; 25 μg of protein were prepared for each samples. Primary antibodies to EGFR (#2232), MET (#3127), pY1234⁄Y1235-MET (#3129), Y1289-Her3 (#4791), pT705-STAT3 (#9131), pS536-NFκB-p65 (#3031), pS465/467-SMAD2 (#3101), Akt (#9272), pS473-Akt (#9271), Erk (#9102), pT202/T204-Erk (#4377), and PARP (#9542) were obtained from Cell Signaling (Beverly, MA). Primary antibodies to pY1068-EGFR (#44-788G) were obtained from Invitrogen. Primary antibodies to Vimentin (BDB550513) were purchased from BD Biosciences (San Diego, CA). Primary antibodies to ZEB1 (sc25388), fibronectin (sc29011), Her3 (sc285), E-cadherin (sc8426), N-cadherin (sc7939), PTEN (sc7974), Snail (sc28199), Slug (sc15391), Twist (sc6269), and Lamin A/C (sc20681) were obtained from Santa Cruz Biotechnology (Santa Cruz, CA). Primary antibodies to β-actin (SigmaA-1978) were purchased from Sigma-Aldrich. Horseradish peroxidase-conjugated goat anti-mouse (NXA931) and anti-rabbit (NA934V) secondary antibodies were obtained from Amersham Biosciences (Piscataway, NJ).

### Establishment of HCC4006ER cells with stable Her3 overexpression

A green fluorescent protein (GFP) retroviral plasmid was generously provided by Dr. Florian Grebien and Dr. Oliver Hantschel (CeMM Institute, Vienna, Austria). Subcloning of Her3 into retroviral plasmid and establishment of stable cell lines with overexpression were performed as previously described [36,38]. In brief, human Her3 cDNA was purchased from Addgene (Cambridge, MA) and amplified by PCR using the forward primer 5’-CACCATGAGGGCGAACGACGCTCT-3’ and reverse primer 5’-CGTTCTCTGGGCATTAGCCTT-3’. The Her3 PCR product was inserted into pENTR D-TOPO vector and then introduced into the pfMSCV C-Strep-HA IRES GFP-GW vector by Gateway LR ClonaseTM II Enzyme Mix Kit (Invitrogen). Retroviruses were packaged in Phoenix HEK293 cells from ATCC (Manassas, VA) and used to infect HCC4006ER cells. Two weeks after infection, GFP-positive HCC4006ER cells were sorted by FACSVantage (BD Biosciences) in the Moffitt Flow Cytometry Core.

### Migration (Scratch) assay and photomicroscopy

HCC4006 and HCC4006ER cells were plated onto 6-cm dishes and incubated overnight in RPMI medium with 10% FBS to create confluent monolayers. The cell monolayers were scraped in a straight line with a 1000-μL pipette tip and incubated for an additional 12 hours. Cell appearance was viewed with an Olympus CKX41 inverted microscope (Olympus, Center Valley, PA) and photographed with an Infinity One camera with Infinity Capture/Analyze software (Lumenera Corp., Ottawa, ON).

### TGF-β1 ELISA

HCC4006 or HCC4006ER cells (5 x 10^5^) were seeded in 6-well plates and incubated overnight in RPMI with 10% FBS. Medium was replaced with serum-free RPMI with or without erlotinib (1 μM) and incubated for an additional 24 hours. Supernatants were collected for ELISA for human TGF-β1 (R&D Systems) following the manufacturer’s recommended protocol.

### Plasmids and liposome-mediated gene transfer

The p3TP-Lux luciferase reporter contains three repeats of the TPA-responsive element fused to a portion of the PAI-1 promoter (provided by J. Massagué, Sloan Kettering Cancer Center, New York, NY). pSBE4-Luc contains four copies of the Smad-binding element (SBE). Transient transfections were performed as described previously [12] with some modifications. Briefly, plasmid DNA (1.7 μg of a luciferase reporter plasmid) was mixed with 10 μL of Fugene reagent (Roche Diagnostic) and incubated for 15 minutes at room temperature before addition to semiconfluent cell cultures in 60-mm tissue culture dishes. Four hours after transfection, cells were treated with either 5 ng/mL TGF-β, 1 μM erlotinib, or both. Forty-eight hours after transfection, the amount of luciferase enzyme activity in cell extracts was determined using the Luciferase Assay System (Promega Corporation). Twenty microliters of cell extracts were added to 100 μL of Luciferase reagent, and the amount of light produced was measured using a Barthold Luminometer (Wallac, Inc., Gaithersburg, MD). The amount of protein present in the cell extracts was determined using the Bio-Rad Bradford assay.

### RNA isolation and purification

Cells were incubated in RPMI medium with 10% FBS for 48 hours at ~80–90% of cell confluence. Total RNA was collected via RNeasy Mini Kit (Qiagen) following the product manual. To ensure high-quality mRNA for gene expression microarray, HCC4006 and HCC4006ER total RNA was examined by Bioanalyzer in the Moffitt Tissue Core.

### Gene expression microarray and bioinformatics analysis

For each cell line, total RNA from triplicate samples was prepared and mixed equally. RNA samples (10 μg for each cell line; 500 ng/μL) were analyzed by Affymetrix Gene Profiling Array chip HG-U133 (Affymetrix, Santa Clara, CA) in the Moffitt Microarray Core. Data were normalized using MAS5, and differences in fold-change were calculated between HCC4006ER and HCC4006 cells (HCC4006ER/HCC4006). A positive fold-change indicated a higher ratio in HCC4006ER cells, whereas a negative fold-change indicated higher expression in HCC4006 cells. Changes in gene expression ±1.5-fold were considered significant [39]. Gene sets were analyzed by MetaCore (http://portal.genego.com; MetaCore, CA) [40]. Network data were integrated and visualized by Cytoscape (http://cytoscape.org/) [41]. Our microarray dataset was submitted to Gene Expression Omnibus (GEO) with the accession number GSE71587.

### Quantitative real-time RT-PCR analysis

After isolation of RNA as above, quantitative real-time RT-PCR was performed as previously described [14,42]. Previously reported primers were used for ZEB1, Snail, Slug, E-cadherin, EpCAM, ESRP1, ST14, vimentin, N-cadherin, and FGFR1 [14].

### ZEB1 overexpression in H358 cells

The ZEB1 gene was cloned into pcDNA5-FRT-TO and transfected into H358-FlpIn TRex cells followed by selection for resistance to 5 μg/mL blasticidin and 100 μg/mL hygromycin, as previously described [14]. 6-myc-ZEB1 overexpression was induced in stably transfected cells using 10 ng/mL doxycycline for 5 days.

### Immunohistochemical analysis of ZEB1

Immunohistochemical staining for ZEB1 was performed as previously described [14]. Briefly, paraffin-embedded tumor slices were deparaffinized in Histoclear and rehydrated followed by antigen retrieval in boiling Antigen Unmasking Solution. The tumor samples had been collected from surgically resected specimens, as reported previously [10]). The institutional review board’s approved written informed consents for the use of tumor tissue specimens were obtained from all patients or from their legal guardians (Institutional Review Board of the University of Occupational and Environmental Health, Japan; IRB number 08–05). Endogenous peroxidases were inhibited (0.3% H_2_O_2_) and tissues were permeabilized with 0.5% Triton (20 minutes). Slides were blocked with 3% BSA/5% goat serum, incubated for 2 hours with primary rabbit anti-ZEB1 (1:50), and washed extensively. Slides were then incubated for 1 hour with biotinylated anti-rabbit antibodies, washed, and developed with Vectastain ABC, according to the manufacturer's protocol (Vector Laboratories, Inc.).

### Quantitation of miR-200c

Total RNA was extracted by TRIzol (Invitrogen). Reverse transcription for mature miR-200c and RNU6B was performed with 20 ng total RNA with the TaqMan MicroRNA reverse transcription kit that includes the MuLV reverse transcriptase (Applied Biosystems, Foster City, CA). The corresponding TaqMan MicroRNA assay was used for quantitative real-time PCR with the GeneAmp 7500 system (Applied Biosystems). Data are expressed as the percent of RNU6B, 100x2^-ΔCt^, where ΔCt = Ct _miR-200c_- Ct _RNU6B_.

### Transfection of short-interfering RNA

Pre-validated short-interfering RNA (siRNA; Invitrogen, catalog no. HSS110549) were used to inhibit endogenous ZEB1. ON-TARGET plus Non-Targeting negative control pools were obtained from Dharmacon (Lafayette, CO) and used as negative control. Transfection was performed with Lipofectamine RNAiMAX from Invitrogen using the reverse transfection procedure as recommended by the manufacturer.

## Results

### Chronic erlotinib exposure of HCC4006 cells generates stable cell-autonomous resistance to EGFR-TKIs without T790M or *MET* amplification

**Fig 1:**
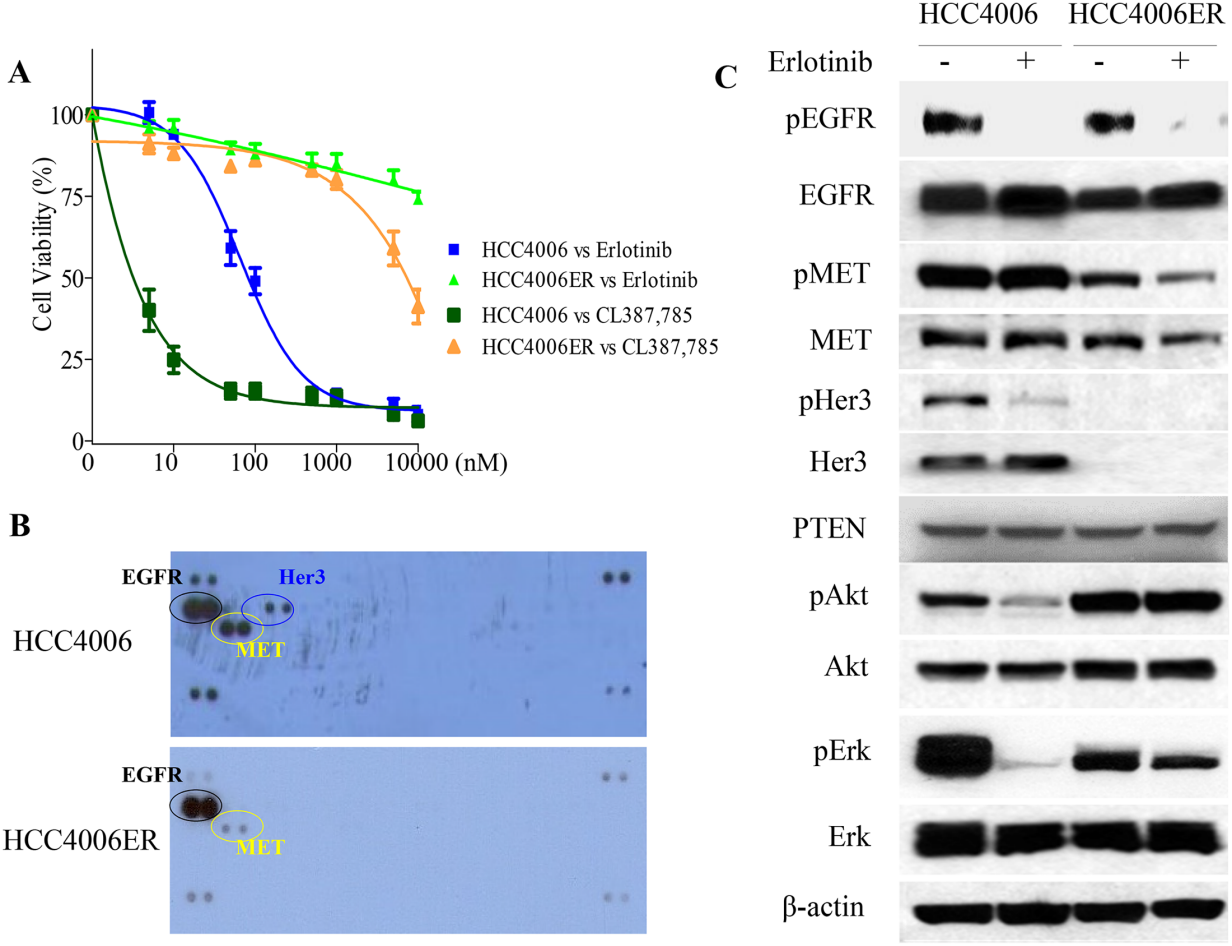
Characterization of HCC4006ER cells, which are highly resistant to erlotinib or the irreversible EGFR-TKI CL387,785. A, HCC4006 and HCC4006ER cells were treated for 72 hours with increasing concentrations of erlotinib or CL387,785. Data generated by cell viability assay (CellTiter-Glo) are expressed as a percentage of the value for untreated cells. The error bars represent SEM of 3 independent experiments. B, HCC4006 and HCC4006ER cells were incubated for 24 hours at ~80–90% of cell confluence. Whole cell lysate of each cell line was collected and subjected to Proteome Profiler Human Phospho-RTK Array Kit to examine the phosphorylation levels of multiple RTKs. Detected phospho-RTKs on the array are circled. The spots in the four corners of the RTK array are positive controls. C, HCC4006 and HCC4006ER cells were incubated for 6 hours ± erlotinib (1 μM). Cell lysates were subjected to protein expression analysis with antibodies to pEGFR, EGFR, pMET, MET, pHer3, Her3, PTEN, pAkt, Akt, pErk, Erk, and β-actin.

To generate EGFR-TKI-resistant clones from an *EGFR*-mutant NSCLC cell line, we exposed EGFR-TKI-sensitive HCC4006 cells with *EGFR* mutation (exon 19; L747-A750del insP) to increasing concentrations of erlotinib (up to 4 μM) for 3 months. HCC4006ER cells became highly resistant to both erlotinib and the irreversible EGFR-TKI, CL387,785 (Fig 1A). This resistance was stable, as it was not reversed by culturing HCC4006ER cells for up to 6 months in erlotinib-free medium (S1 Fig). We also established 5 single cell clones of HCC4006ER cells (HCC4006ER-S1 to -S5 cells), which were each individually resistant to erlotinib to the same degree as the bulk HCC4006ER population (S2A Fig). Thus, the resistant phenotype was stable and cell autonomous.

We examined the *EGFR* gene status of both sensitive and resistant cells for the secondary T790M mutation in exon 20. Although HCC4006ER cells retained exon 19 L747-A750del insP, T790M was not detected even with the PCR-invader assay [34], which is more sensitive than direct sequencing (data not shown). In addition, we did not find *KRAS* hotspot mutations (exon 1 G12V and exon 2 Q61H; data not shown), which is associated with primary EGFR-TKI resistance in NSCLC [43]. To investigate other reported mechanisms of EGFR-TKI resistance such as *MET* amplification [3], activation of IGFR signaling [44], or loss of PTEN protein [45], we examined key molecules of the EGFR pathway by Western blot and employed a phospho-RTK array to survey for various activated RTKs. However, we observed no previously identified resistance mechanism to EGFR-TKI, including upregulated MET or IGFR activity (Fig 1B) or loss of PTEN protein in HCC4006ER cells (Fig 1C). In terms of EGFR signaling, the ability of erlotinib to inhibit EGFR phosphorylation was retained in both HCC4006 and HCC4006ER cells. However, phospho-Akt and Erk levels were sensitive to erlotinib only in the parental HCC4006 cells, in contrast to HCC4006ER cells (Fig 1C).

**Fig 2:**
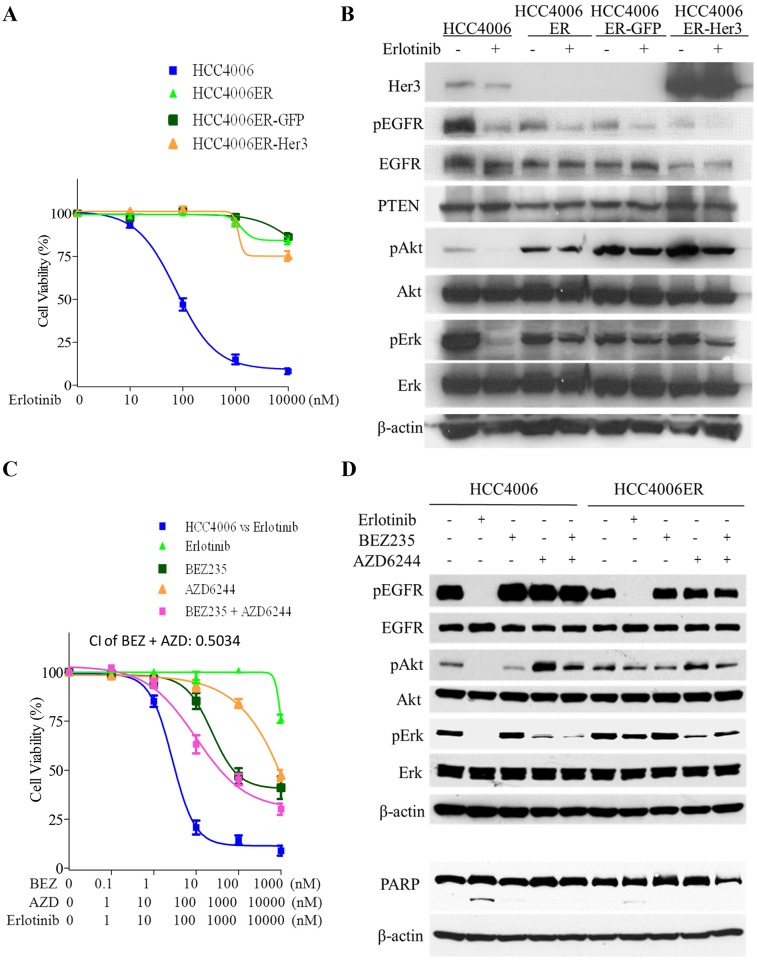
Neither Her3 loss nor persistent activation of pAkt and pErk is essential for the resistance in HCC4006ER cells. A, HCC4006ER cells with stable GFP or Her3 overexpression (HCC4006ER-GFP and HCC4006ER-Her3 cells) as well as HCC4006 and the original HCC4006ER cells were treated for 72 hours with increasing concentrations of erlotinib. Data generated by cell viability assay (CellTiter-Glo) are expressed as a percentage of the value for untreated cells. The error bars represent SEM of 3 independent experiments. B, HCC4006, HCC4006ER, HCC4006ER-GFP, and HCC4006ER-Her3 cells were incubated for 6 hours ± erlotinib (1 μM). Cell lysates were subjected to protein expression analysis with antibodies to Her3, pEGFR, EGFR, PTEN, pAkt, Akt, pErk, Erk, and β-actin. C, HCC4006ER cells were treated for 72 h with increasing concentrations of erlotinib alone, BEZ235 alone, AZD6244 alone, or BEZ235 and AZD6244 in combination. HCC4006 cells were treated for 72 hours with increasing concentrations of erlotinib for 72 hours to plot a reference curve. Data generated by cell viability assay (CellTiter-Glo) are expressed as a percentage of the value for untreated cells. The error bars represent SEM of 3 independent experiments. Combination index (CI) at IC50 dose of BEZ235 combined with AZD6244 was calculated by CompuSyn software. CI>1, CI = 1, and CI<1 indicate antagonistic, additive, and synergistic effects, respectively. D, Both HCC4006 and HCC4006ER cells were incubated for 6 or 24 hours ± erlotinib (1 μM), BEZ235 (500 nM), or AZD6244 (1 μM) as indicated. Cell lysates were subjected to protein expression analysis with antibodies to pEGFR, EGFR, pAkt, Akt, pErk, and Erk (samples of 6 hours) or to PARP along with antibodies to β-actin as a loading control (samples of 24 hours).

A previous study reported that Her3 mediates PI3K/Akt pathway signaling in gefitinib-sensitive NSCLC cell lines [46]. However, our Western blot analyses demonstrated that HCC4006ER cells completely lost Her3 protein expression compared with HCC4006 cells, explaining the loss of Her3 phosphorylation in these cells observed in the RTK array and Western analysis (Fig 1B and 1C). To determine whether HER3 loss could explain resistance to erlotinib in HCC4006ER cells, we generated Her3 overexpressing cells (HCC4006ER-Her3 cells) using lentiviral infection to examine the effects of erlotinib on cell proliferation and the EGFR pathway. HCC4006ER-Her3 cells maintained their resistance to erlotinib (Fig 2A), as well as persistence of phospho-Akt and phospho-Erk (Fig 2B), similar to the original HCC4006ER cells and control lentivirus-derived GFP overexpression (HCC4006ER-GFP). These results indicate that Her3 loss does not drive erlotinib resistance in HCC4006ER cells.

With persistent phosphorylation of Akt and Erk in HCC4006ER cells, we examined the effects of the PI3K/mTOR inhibitor, BEZ235, and the MEK inhibitor, AZD6244. We observed that phospho-Akt was activated by phospho-Erk inhibition with AZD6244 in both HCC4006 and HCC4006ER cells. This phospho-Akt activation induced by AZD6244 was not completely inhibited by BEZ235 in HCC4006 and HCC4006ER cells (Fig 2D). The combination of BEZ235 and AZD6244 did not restore the inhibition of cell proliferation or PARP cleavage in HCC4006ER cells induced by erlotinib treatment in HCC4006 cells, although Combination Index (CI) of this combination indicates synergistic effect (CI<1) (Fig 2C and 2D). These results demonstrate that the dual inhibition of Akt and Erk did not overcome erlotinib resistance in HCC4006ER cells, although phospho-Akt and Erk were persistently activated independent of EGFR pathway.

### HCC4006ER cells show an EMT phenotype with activation of the TGF-β/SMAD pathway

**Fig 3:**
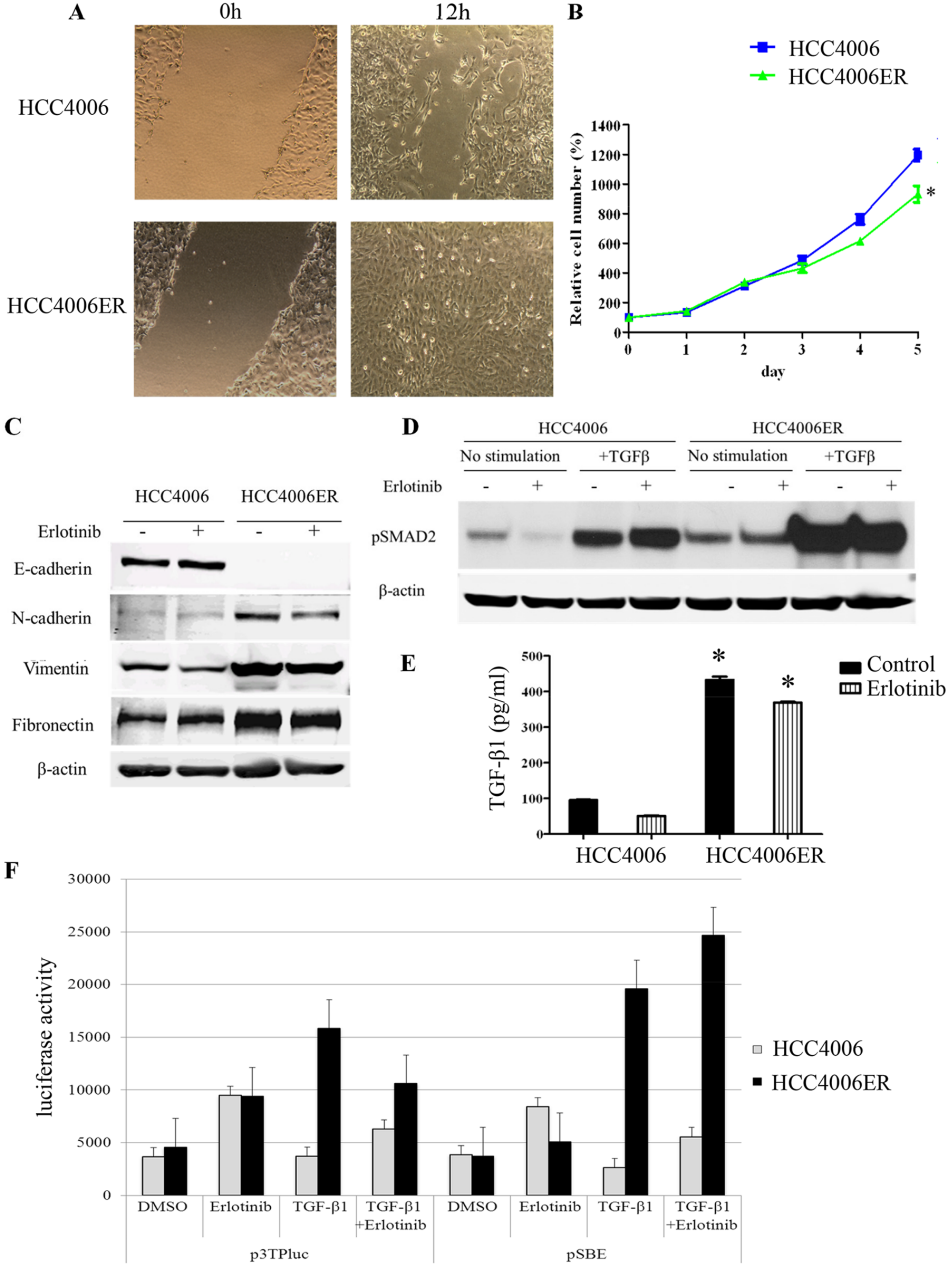
HCC4006ER cells have EMT-phenotype with activation of TGF-β/SMAD signaling. A, Monolayers of HCC4006 and HCC4006ER cells were scraped in a straight line with a 1000-μL pipette tip. Monolayer photos with scratches were taken after 12-hour incubation. B, HCC4006 and HCC4006ER cells were plated at 1x10^3^ cells/well in black wall 96-well plates, incubated in RPMI with 10% FBS, and allowed to grow for indicated days. Data generated by cell viability assay (CellTiter-Glo) are expressed as a percentage of the value for untreated cells. Determinations were done in triplicate. Bars, SEM. *P < 0.0001 for HCC4006 versus HCC4006ER (Student *t* test). C, HCC4006 and HCC4006ER cells were incubated for 6 hours ± erlotinib (1 μM). Cell lysates were subjected to protein expression analysis with antibodies to E-cadherin, N-cadherin, vimentin, fibronectin, and β-actin. D, HCC4006 and HCC4006ER cells were incubated for 6 hours ± TGF-β1 (10 ng/ml) or erlotinib (1 μM), as indicated. Cell lysates were subjected to protein expression analysis with antibodies to pSMAD2 and to β-actin. E, HCC4006 or HCC4006ER cells were incubated overnight. Medium was replaced with serum-free RPMI with or without erlotinib (1 μM) and incubated for an additional 24 hours. Supernatants were collected and subjected to TGF-β1 ELISA. Determinations were done in triplicate. Bars, SEM. *P < 0.0001 versus HCC4006 cells with or without erlotinib treatment (Student *t* test). F, TGF-β-induced transcription is increased by co-treatment with TGF-β and erlotonib in transient and transfections with TGF-β-responsive luciferase constructs. HCC4006 and HCC4006ER cells were transiently transfected with p3TP-Lux reporter or pSBE4 and treated with DMSO, 5 ng/mL TGF-β1, 1 μM erlotinib, or a combination of 5 ng/mL TGF-β1 and 1 μM erlotinib for 48 hours. After 48 hours, luciferase activity was determined as described in Materials and Methods.

To determine whether resistance in HCC4006ER cells was associated with an EMT process, we tested cell migration using scratch assays. In HCC4006ER cells, the gap was completely closed within 12 hours, while more than 12 hours were required in the parental HCC4006 cells (Fig 3A). We also found that proliferation of HCC4006ER cells was significantly slower than parental cells (Fig 3B), consistent with recent studies suggesting slower growth of drug-resistant cells [47]. Several reports have indicated that EMT is associated with primary and acquired resistance to EGFR-TKI in NSCLC [4–11]. The increased migration, erlotinib resistance, and reduced proliferation of HCC4006ER cells were consistent with an EMT process. To further examine this possibility, we looked for EMT-related molecules in HCC4006 and HCC4006ER cells. Importantly, we found loss of E-cadherin and upregulation of N-cadherin, vimentin, and fibronectin in HCC4006ER cells, which were not affected by erlotinib treatment (Fig 3C). Moreover, we found that all single cell-resistant clones (HCC4006ER-S1 to -S5) had undergone EMT, as well as loss of Her3 protein, similar to the results observed in the original HCC4006ER cells (S2B Fig). Previous study indicated that the maintenance of gefitinib prevented the EMT process and inhibited cell migration in gefitinib-resistant NSCLC cells with *MET*-amplification (but without EMT phenotype) [48]. However, the expression levels of EMT markers (E-cadherin, N-cadherin, vimentin, and fibronectin) were not affected by continues exposure of erlotinib for 72 hours in HCC4006ER cells (S3A Fig). In addition, the ability of migration in HCC4006ER cells were still superior to that in HCC4006 cells even incubated with erlotinib (S3B Fig). These results suggested that EMT phenotype in HCC4006ER cells were stable regardless of the presence of erlotinib.

Consistent with previous studies showing that the TGF-β pathway is critical for EMT-related acquired EGFR-TKI resistance in NSCLC [8,9], we found higher basal levels of SMAD2 phosphorylation in HCC4006ER cells. As expected, SMAD2 phosphorylation further increased after TGF-β treatment, and this increase was independent of erlotinib treatment (Fig 3D). In addition, compared to HCC4006 cells, the amount of TGF-β1 in the medium was upregulated in HCC4006ER cells (independent of erlotinib treatment) (Fig 3E), suggesting that the TGF-β pathway was activated secondary to increased ligand expression. Activation of the TGF-β pathway in HCC4006ER cells was confirmed by transfection of the TGF-β-responsive luciferase reporter plasmids, pSBE4 and p3TPlux, into both HCC4006 and HCC4006ER cells treated with 5 ng/mL TGF-β and/or 1 μM erlotinib. As can be seen in Fig 3F, TGF-β-driven transcription is higher in HCC4006ER cells than in parental cells when stimulated by TGF-β regardless of the presence of erlotinib (Fig 3F). These results suggest that the TGF-β pathway is activated independently of EGFR pathway in HCC4006ER cells compared with parental HCC4006 cells.

### Inhibitors of EMT-related pathways are insufficient to resensitize HCC4006ER cells to erlotinib

**Fig 4:**
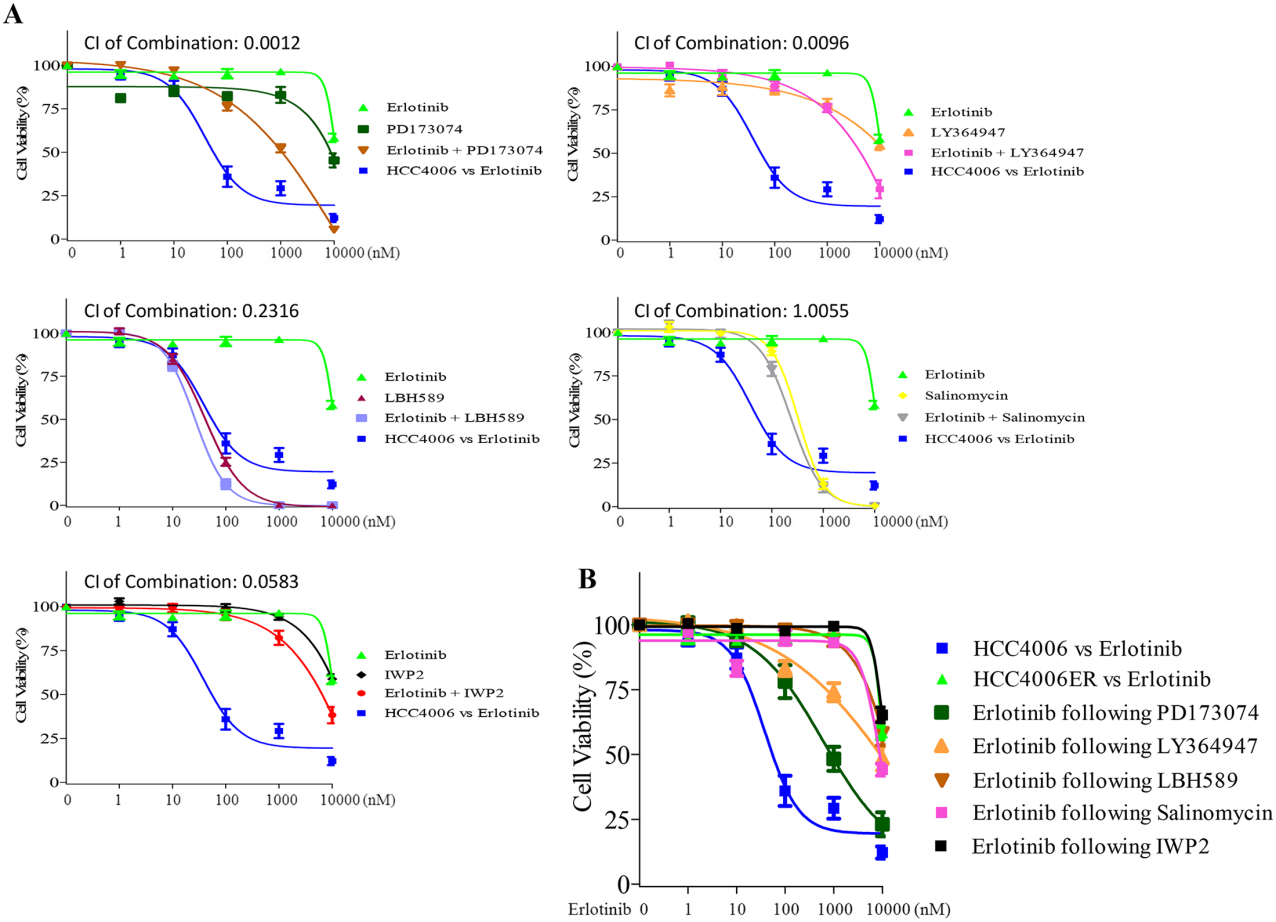
Effects of reagents against EMT-related pathways on cell growth in HCC4006ER cells. A, HCC4006ER cells were treated for 72 hours with increasing concentrations of indicated reagents ± erlotinib. HCC4006 cells were treated for 72 hours with increasing concentrations of erlotinib for 72 hours to plot a reference curve. Data generated by cell viability assay (CellTiter-Glo) are expressed as a percentage of the value for untreated cells. The error bars represent SEM of 3 independent experiments. Combination index (CI) at IC50 dose of each combination treatment was calculated by CompuSyn software. CI>1, CI = 1, and CI<1 indicate antagonistic, additive and synergistic effects, respectively. B, HCC4006ER cells were treated for 72 hours with increasing concentrations of erlotinib, following treatment of PD173074 (1 μM), LY364947 (1 μM), LBH589 (10 nM), salinomycin (100 nM), or IWP2 (1 μM) for 14 days. Data generated by cell viability assay (CellTiter-Glo) are expressed as a percentage of the value for untreated cells. The error bars represent SEM of 3 independent experiments.

FGFR [6,12], TGF-β [8,9], and WNT pathways [13] are thought to be involved in EMT. In addition to inhibitors of these pathways, several reagents, including histone deacetylase (HDAC) inhibitors [9,49] and salinomycin [50], have been suggested to reverse EMT in other systems. We applied inhibitors of FGFRs (PD173074), TGF-β R1 (LY364947), HDACs (LBH589), the WNT pathway (IWP2), as well as the potassium ionophore and cancer stem cell inhibitor salinomycin, with or without erlotinib, to HCC4006ER cells. Although LBH589 and salinomycin inhibited cell growth as single agents, no additive effect (CI>1) of salinomycin combined with erlotinib was observed in HCC4006ER cells (Fig 4A). LY364947 and IWP2 failed to inhibit cell growth with or without erlotinib despite their synergistic effects (CI<1) combined with erlotinib (Fig 4A). The FGFR inhibitor PD173074 slightly resensitized HCC4006ER cells to erlotinib with synergistic effect (CI<1); however, it did not cause reversion to the level of parental HCC4006 cells (Fig 4A). In addition, the IC_50_ values of all these non-EGFR reagents in HCC4006ER cells were similar to those in parental HCC4006 cells (S1 Table).

We also examined erlotinib sensitivity following prolonged treatment with these compounds for 14 days. Although we observed slight improvement of erlotinib sensitivity when treated with PD173074, none of the compounds was able to completely resensitize HCC4006ER cells to erlotinib (Fig 4B). In addition, an anti-IL-6 monoclonal antibody, CNTO328, was tested based on the report demonstrating that the IL-6/TGF-β axis is critical for EGFR-TKI-resistant H1650 NSCLC cells with EMT phenotype [8]. However, IL-6 inhibition with CNTO328 did not inhibit cell growth in HCC4006ER cells, even when combined with erlotinib at clinically relevant dose ranges (S4 Fig). These results indicate that these EMT-related inhibitors are insufficient to resensitize HCC4006ER cells to erlotinib, suggesting that erlotinib resistance in HCC4006ER is regulated by multiple EMT pathways or other mechanisms.

### *ZEB1* is a key regulator of genome alterations in HCC4006ER cells

**Fig 5:**
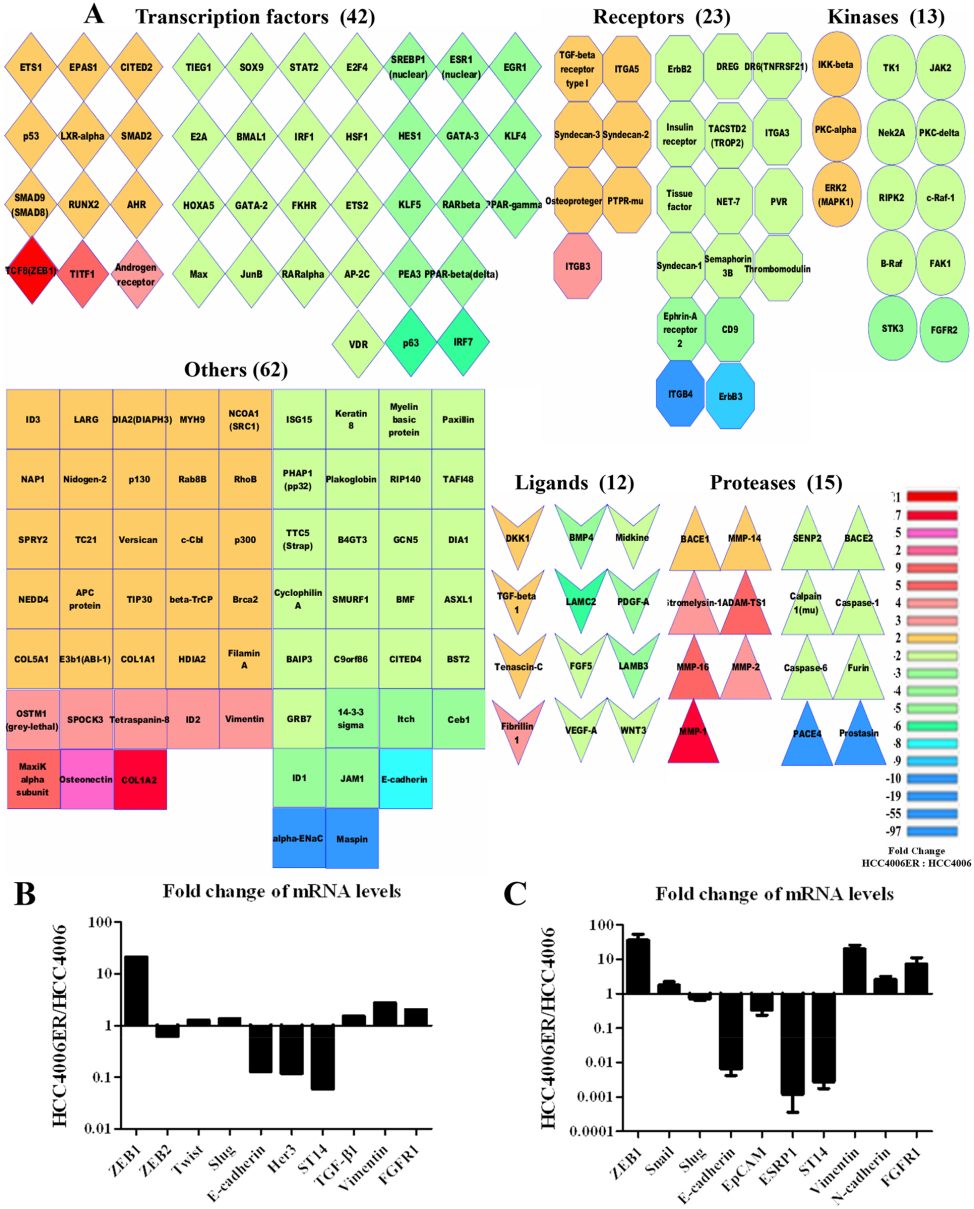
Gene expression microarray revealed that ZEB1 is a critical gene in HCC4006ER cells. A, An enrichment and classification analyses using our microarray results were performed by MetaCore and Cytoscape. On the heatmap, 167 genes were selected as important genes in HCC4006ER cells by setting the cutoff at ≥7 connections in the whole network. The color represents the fold-change (HCC4006ER/HCC4006) by comparing the intensity of gene probes from our microarray. B, Representative ZEB1-responsive genes or EMT-related genes were selected from our microarray to show the fold-change (HCC4006ER/HCC4006) in the bar graph. C, mRNA levels of indicated ZEB1 responsive genes in HCC4006 and HCC4006ER cells were measured by quantitative real-time RT-PCR. Values are expressed as fold-change between HCC4006 and HCC4006ER cells (HCC4006ER/HCC4006). Determinations were done in triplicate. Bars, SD.

We examined global patterns of gene expression using Affymetrix arrays in HCC4006 and HCC4006ER cells. Approximately 4000 genes were differentially expressed between the two cell lines (HCC4006ER:HCC4006; cutoff set as ≥ 1.5 or ≤ -1.5). Pathway enrichment analysis implicated the TGF-β/SMAD, WNT, ephrin, and integrin signaling pathways, which are related to cell adhesion, migration, and EMT (S2 Table). To identify important regulators of those gene sets, we constructed global biological networks surrounding the ~4000 differentially regulated genes using gene-gene interactions curated by GeneGO. We searched for genes highly connected with other genes (cutoff set as ≥7 connections) to identify potential “hubs” acting as global regulators [51]. We identified 167 highly connected genes (Fig 5A), which were further classified by gene type/function and colored according to fold-change levels (HCC4006ER:HCC4006) on the pseudo-heatmap (Fig 5A). As shown in the genes of transcriptional factors on our heatmap, *ZEB1* (*TCF8*), a known regulator of EMT [14], was more than 20 times higher in HCC4006ER cells than in HCC4006 cells (Fig 5A). Other EMT-related transcription factors, such as *ZEB2* (*SIP1*), *TWIST1* (*Twist*), and *SNAI2* (*Slug*), were not present on the heatmap because of their minor change in expression (fold-change of -1.60, 1.27, and 1.35, respectively; Fig 5B). The loss of Her3 and E-cadherin, as well as increased TGF-β1 and vimentin, was also confirmed at the mRNA level (Fig 5A and 5B).

We recently identified *ZEB1*-regulated genes using an Affymetrix-based expression database of 38 NSCLC cell lines [14]. These *ZEB1*-responsive genes were examined in the microarray data to identify alterations between HCC4006ER and HCC4006 cells (S3 and S4 Tables). Along with *ZEB1* upregulation in HCC4006ER cells, 212 of 220 genes negatively correlated with *ZEB1*, such as *CDH1* (*E-cadherin*), *ERBB3* (*Her3*), and *ST14*, were also negatively correlated with *ZEB1* in our microarray data (S3 Table and Fig 5B). Similarly, 70 of 75 *ZEB1* positively correlated genes, such as *Vimentin* and *FGFR1*, were also positively correlated with *ZEB1* in our data set (S4 Table and Fig 5B). These results suggest that *ZEB1* is critical for the genome alterations enabling transition from HCC4006 to HCC4006ER cells.

To validate our microarray results, we tested selected genes either negatively correlated with *ZEB1* (*E-cadherin*, *EpCAM*, *ESRP1*, and *ST14*) or positively correlated with *ZEB1* (*Vimentin* and *FGFR1*) [14] and analyzed them along with *ZEB1*, *SNAI1* (*Snail*), *SNAI2* (*Slug*), and *N-cadherin* using quantitative RT-PCR. The mRNA level of ZEB1 in HCC4006ER cells was more than 30 times higher than in HCC4006 cells, whereas fold-changes (HCC4006ER/HCC4006) of *SNAI1* (*Snail*) and *SNAI2* (*Slug*) were 1.8 and 0.7, respectively (Fig 5C). As expected, mRNA levels of E-cadherin, EpCAM, ESRP1, and ST14 were downregulated, while those of vimentin, N-cadherin, and FGFR1 were upregulated in HCC4006ER cells (Fig 5C). In addition, ZEB1 protein was upregulated in nuclear extracts of HCC4006ER cells, although no apparent changes in other EMT-related transcription factors were observed, except for the loss of STAT3 activation in HCC4006ER cells (S5 Fig).

### ZEB1 knockdown reverses EMT phenotype and erlotinib sensitivity in HCC4006ER cells

**Fig 6:**
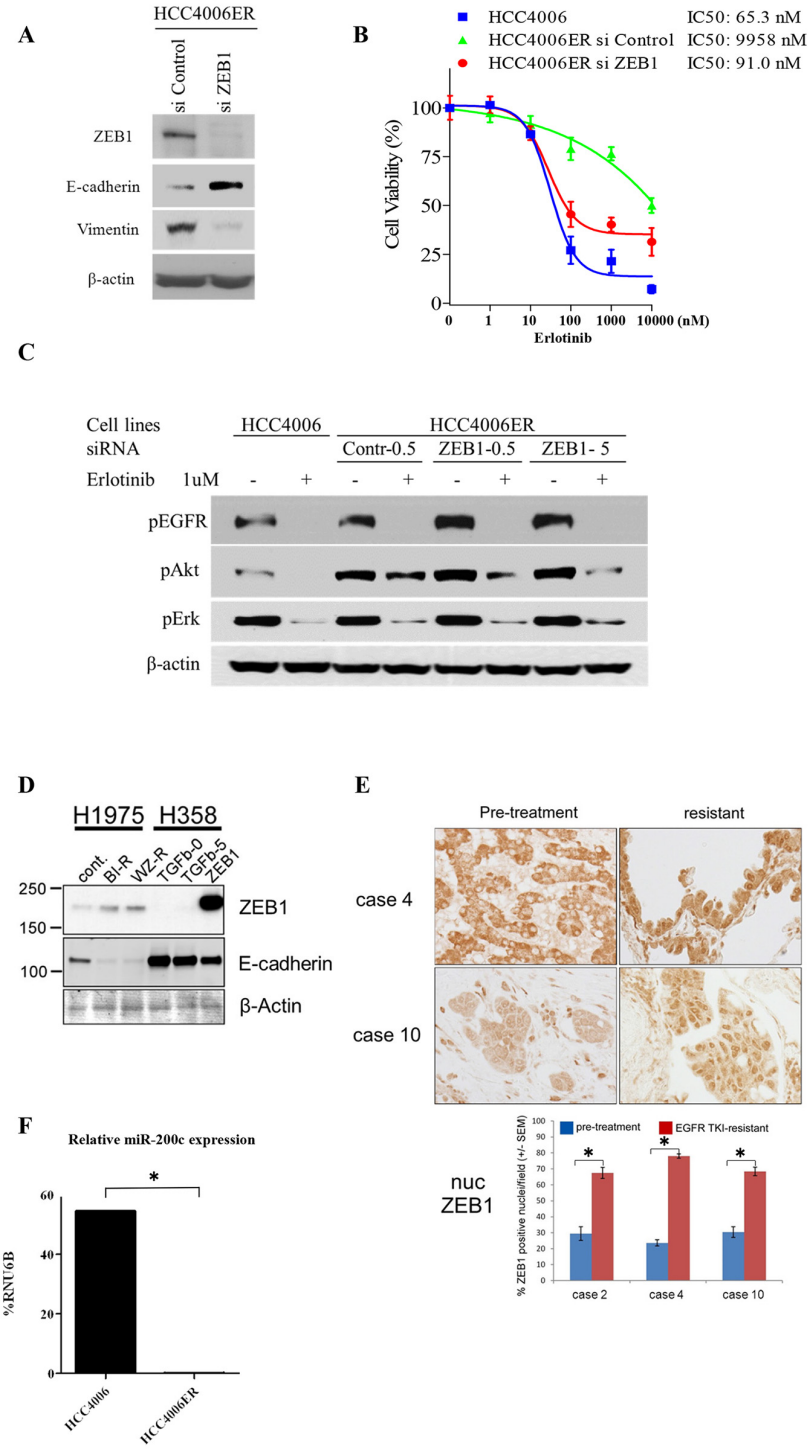
Effects of knockdown of ZEB1 or overexpression of miR-200c on EMT markers and erlotinib sensitivity in HCC4006ER cells. A, HCC4006ER cells were plated at 3x10^5^ per well in 6-well plate and transfected with ZEB1 or control siRNA at 5 nM as a final concentration. Cells were harvested for analysis at 96 hours post-transfection. Cell lysates were subjected to protein expression analysis with antibodies to ZEB1, E-cadherin, vimentin, and β-actin. B, HCC4006ER cells were plated at 3x10^3^ cells/ well in black wall 96-well plate, transfected with ZEB1 or control siRNA at 5 nM as a final concentration, and treated for 72 hours at 48 hours after siRNA transfection with increasing concentrations of erlotinib. HCC4006 cells were treated for 72 hours with increasing concentrations of erlotinib for 72 hours to plot a reference curve. Data generated by cell viability assay (CellTiter-Glo) are expressed as a percentage of the value for erlotinib-untreated cells with control siRNA. Control siRNA or transfection reagent did not affect cell viability in HCC4006ER cells at this concentration. The error bars represent SEM of 3 independent experiments. C, HCC4006 and HCC4006ER cells were plated at 3x10^5^ per well in 6-well plate. HCC4006ER cells were transfected with ZEB1 or control siRNA at 0.5 or 5 nM as indicated. HCC4006 cells and siRNA-transfected HCC4006ER cells were incubated for 6 hours ± erlotinib (1 μM) at 48 hours after siRNA transfection. Cell lysates were subjected to protein expression analysis with antibodies to pEGFR, pAkt, pErk, and β-actin. D, Cell lysates of H1975, H1975 BIBW-R, and H1975 WZ-R cells were subjected to protein expression analysis with antibodies to ZEB1, E-cadherin, and β-actin. H358 cells, with and without 5 ng/mL TGF-β treatment for 48 hours or with ZEB1 overexpression, served as positive controls (see Materials and Methods for cell line details). TGF-β stimulation or ZEB1 overexpression reduced E-cadherin expression in H358 cells, as expected. E, Tumor samples from NSCLC patients who were treated with EGFR-TKI and developed resistance accompanied by an EMT phenotype (cases 2, 4, and 10 in our previous report (10); case 2 had T790M and PTEN loss in addition to EMT, whereas cases 4 and 10 did not have other known resistant mechanism after EGFR-TKI treatment). ZEB1 immunostaining analysis was done before and after EGFR-TKI treatment. Representative ZEB1 immunostaining images from cases 4 and 10 (before and after EGFR-TKI treatment) are shown in the upper panel. Percentage of ZEB1-positive nuclei per field was demonstrated for each case (before and after EGFR-TKI treatment) in the bottom panel. Determinations were done in triplicate. Bars, SEM. *P < 0.00001 for before versus after EGFR-TKI treatment (Student *t* test). F, Expression levels of miR-200c and RNU6B in HCC4006 and in HCC4006ER cells were measured by the quantitative real-time PCR with the corresponding TaqMan MicroRNA assay. Values are expressed as % of RNU6B. Determinations were done in triplicate with two machine replicates. *P < 0.005 for HCC4006 versus HCC4006ER (Student *t* test).

Since the gene expression profiling suggested *ZEB1* as a key regulator of EMT in HCC4006ER cells, we examined the effects of ZEB1 knockdown by siRNA on EMT markers in these cells. As expected, we observed upregulation of E-cadherin and downregulation of vimentin along with ZEB1 knockdown (Fig 6A). We further assessed whether ZEB1 knockdown restored erlotinib sensitivity. Strikingly, siRNA targeting ZEB1 resensitized HCC4006ER cells to erlotinib to the same IC_50_ level as HCC4006 cells (Fig 6B). These results, along with coordinated gene expression driven by ZEB1, identify ZEB1 as responsible for both the EMT process and EGFR-TKI resistance in HCC4006ER cells. We next examined the effects of siRNA targeting ZEB1 on EGFR signaling with or without erlotinib in HCC4006 and HCC4006ER cells. Akt was inhibited by erlotinib even in HCC4006ER cells when SiZEB1 was employed, while no such effect was observed on pErk (Fig 6C). These results suggest that pAkt is critical for cell proliferation in HCC4006ER cells.

To determine whether our findings were more broadly applicable to lung tumors with EGFR-activating mutations, we generated H1975 BIBW-R (BIBW2992 resistant) and H1975 WZ-R (WZ4002 resistant) cells by exposing EGFR-TKI-resistant H1975 cells (with exon 21; L858R and exon 20; T790M) to increasing concentrations of the irreversible EGFR-TKI BIBW2992 (afatinib) or the T790M selective EGFR-TKI WZ4002 (up to 3 μM or 15 μM, respectively) for 3 months. H1975 BIBW-R and H1975 WZ-R cells became resistant to both BIBW2992 and WZ4002 (S6 Fig). Of note, when H1975 BIBW-R and H1975 WZ-R cells were compared with the parent H1975, ZEB1 expression was increased and E-cadherin expression was abolished (Fig 6D).

We next examined ZEB1 immunostaining in 3 cases of human NSCLC samples that had acquired EGFR-TKI resistance along with an EMT phenotype (cases 2, 4, and 10 in our previous report [10]). We observed a significant increase in positivity and nuclear localization of ZEB1 in all 3 cases after EGFR-TKI treatment (Fig 6E). These results suggest that ZEB1 overexpression is generally observed in multiple NSCLC cell lines and NSCLC patient samples with EMT-related EGFR-TKI resistance.

Finally, we examined miR-200c levels, which can negatively regulate ZEB1 and related gene expression changes associated with EMT [30–32], in both HCC4006 and HCC4006ER cells. miR-200c expression in HCC4006ER cells was significantly reduced compared with HCC4006 cells (Fig 6F). Together, our results suggest that *ZEB1* overexpression is responsible for EMT-related acquired resistance to EGFR-TKIs in NSCLC, and that miR-200c may contribute to this process.

## Discussion

EMT is essential for tumor invasion, metastasis, and drug resistance in many cancers [29] [16,22,52]. EMT-like changes have been observed in subsets of NSCLC patients with acquired EGFR-TKI resistance [10,11]. Here, we generated HCC4006ER cells that underwent EMT with activation of the TGF-β pathway in the absence of other known mechanisms of EGFR-TKI resistance, such as a T790M gatekeeper mutation or *MET* amplification. Gene expression microarrays and quantitative RT-PCR showed that the mRNA level of ZEB1 was considerably higher in HCC4006ER cells than in control HCC4006 cells, with the absence of significant changes in other EMT-related transcription factors such as ZEB2, Snail, Slug, or Twist. Also striking was the observation of coordinated regulation of *ZEB1*-dependent genes indicating the potential reprogramming of HCC4006 cells by *ZEB1*. Consistent with this idea, knockdown of *ZEB1* reversed the EMT phenotype in HCC4006ER cells, and more importantly erlotinib sensitivity in HCC4006ER cells reverted to the same level as parental HCC4006 cells, suggesting that ZEB1 mediates the resistance in these cells. We also demonstrated that ZEB1 upregulation was observed in additional NSCLC cell lines and human samples with EMT-related EGFR-TKI resistance, suggesting that ZEB1 is a possible target to overcome this kind of resistance in clinical lung cancer. Previous reports have pointed out the importance of miR-200c in the EMT process [30–32]. In line with these studies, we observed that miR-200c expression was markedly reduced in HCC4006ER cells. A recent study has shown that EMT process by miR200/ZEB1 axis results in PD-L1 upregulation or CD8(+) TIL immunosuppression in NSCLC, suggesting that anti-PDL1 therapy is a potential strategy against NSCLC with EMT phenotype [53]. Future studies should examine microRNAs and PD-L1 in TKI acquired resistance cell line models as well as in patients with acquired EGFR-TKI resistance. Thus, we conclude that *ZEB1* mediates both the EMT process and erlotinib resistance in HCC4006ER cells and that *ZEB1* could be regulated by miR-200c in these cells.

Our experiments also highlight potential therapeutic implications. In addition to EMT, we observed complete Her3 loss in HCC4006ER cells, yet re-expression of Her3 in HCC4006ER cells was unable to overcome erlotinib resistance. Loss of Her3 associated with EMT may confer resistance to Her3 targeting agents, such as the anti-Her3 monoclonal antibody MM-121, which is currently being assessed in patients with advanced NSCLC as a phase I/II trial (ClinicalTrials.gov identifier NCT00994123). In addition, we observed that pAkt and pErk levels were persistent despite erlotinib treatment in HCC4006ER cells. However, the dual inhibition of PI3K/Akt and MEK/Erk pathways did not lead to synergistic inhibition of cell proliferation and induce apoptosis in these cells. Our results also suggest dual inhibition of PI3K and MEK pathways might not be particularly useful for EMT-related EGFR-TKI resistance in NSCLC, while this approach may be more effective against T790M-related acquired resistance to EGFR-TKI [54].

We also assessed the effects of several inhibitors against pathways possibly related to the EMT process in HCC4006ER cells. Single-agent HDAC inhibitor (LBH589) and salinomycin retained their activity in HCC4006ER cells, consistent with previous studies showing that these agents are effective in cells with the EMT phenotype [9,49,50]. However, long-term treatment with these inhibitors did not resensitize HCC4006ER cells to erlotinib. It remains unclear how salinomycin, which was found by global drug screening [50], mechanistically acts in cells with the EMT phenotype, whereas HDACs are thought to be directly involved in the EMT process [55,56]. There are several articles showing that epigenetic alteration is critical for EMT process, possibly explaining the efficacy of HDAC inhibitor in HCC4006ER cells [26,27,57–59]. In pre-clinical models, HDAC inhibitors also have efficacy in EGFR-TKI drug-intolerant NSCLC cells caused by KDM5A upregulation [60], suggesting that HDAC inhibitors may have a role in overcoming particular types of EGFR-TKI resistance. Although our pathway enrichment analysis showed that TGF-β and WNT pathways are critical in HCC4006ER cells, neither long-term nor short-term treatment of TGF-β or WNT pathway inhibitors affected either cell viability or erlotinib sensitivity in HCC4006ER cells. It is possible that these inhibitors could prevent EMT, but our results so far do not suggest their ability to reverse EMT [61]. We demonstrated that both long-term and short-term treatment with a FGFR inhibitor slightly improved erlotinib sensitivity in HCC4006ER cells, in line with our microarray and quantitative RT-PCR showing that mRNA of FGFR1 is upregulated in these cells. Other groups have shown that gefitinib-resistant HCC4006 cells with an EMT phenotype were sensitive to the FGFR inhibitor AZ8010, which was dependent on FGFR1 overexpression [12]. However, no reagent that we assessed in HCC4006ER cells was able to completely reverse erlotinib sensitivity to the same degree as parental HCC4006 cells. A previous study reported that IL-6 neutralizing antibody decreased cell viability in the H1650-M3 cell line, which is another model of acquired EGFR-TKI resistance with an EMT phenotype mediated by TGF-β and IL-6/STAT3 pathways [8]. However, neither STAT3 activation nor anti-tumor activity of IL-6 antibody CNTO328 was observed in our HCC4006ER cells, suggesting that the IL-6/STAT3 pathway is not necessarily activated in EMT-related acquired EGFR-TKI resistance in NSCLC. While our results suggest that several agents, including salinomycin, HDAC, and FGFR inhibitors, are candidates against EMT-related acquired resistance to EGFR-TKI, none completely resensitized HCC4006ER cells to erlotinib. Future efforts should be directed toward a better understanding of ZEB1 and novel targeting strategies to resensitize these tumors to EGFR-TKI.

## Supporting Information

S1 Fig HCC4006ER cells maintain their resistance in erlotinib-free condition for 6 months. HCC4006ER cells cultured in erlotinib-free medium for 2 or 6 months, as well as HCC4006 and the original HCC4006ER cells, were treated for 72 hours with increasing concentrations of erlotinib. Data generated by cell viability assay (CellTiter-Glo) are expressed as a percentage of the value for untreated cells. The error bars represent SEM of 3 independent experiments.
(PPTX)

S2 Fig Single cell clones of HCC4006ER cells show erlotinib resistance with EMT phenotype similar to HCC4006ER cells. A, Single cell clones of HCC4006ER cells (HCC4006ER-S1 to -S5 cells) as well as HCC4006 and the original HCC4006ER cells were treated for 72 hours with increasing concentrations of erlotinib. Data generated by cell viability assay (CellTiter-Glo) are expressed as a percentage of the value for untreated cells. The error bars represent SEM of 3 independent experiments. B, Cell lysates of HCC4006, HCC4006ER, and single cell clones of HCC4006ER cells (HCC4006ER-S1 to -S5 cells) were subjected to protein expression analysis with antibodies to E-cadherin, N-cadherin, vimentin, fibronectin, Her3, and β-actin.
(PPTX)

S3 Fig The expression of EMT markers as well as cell migration are not affected by erlotinib exposure in HCC4006ER cells. A, HCC4006 and HCC4006ER cells were incubated for 72 hours ± erlotinib (1 μM). Cell lysates were subjected to protein expression analysis with antibodies to E-cadherin, N-cadherin, vimentin, fibronectin, and β-actin. B, Monolayers of HCC4006 and HCC4006ER cells were scraped in a straight line with a 1000-μL pipette tip. Monolayer photos with scratches were taken after 12-hour incubation with erlotinib (1 μM).
(PPTX)

S4 Fig Effects of the anti-IL-6 monoclonal antibody CNTO328 on cell growth in HCC4006ER cells. HCC4006ER cells were treated for 72 hours with increasing concentrations of erlotinib alone, CNTO328 alone, or erlotinib and CNTO328 in combination. Data generated by cell viability assay (CellTiter-Glo) are expressed as a percentage of the value for untreated cells. The error bars represent SEM of 3 independent experiments.
(PPTX)

S5 Fig Validation of the results of gene expression microarray using Western blotting. Nuclear extract of both HCC4006 and HCC4006ER cells were subjected to protein expression analysis with antibodies to ZEB1, pT705-STAT3, pS536-NFκB-p65, Snail, Slug, Twist, and Lamin A/C.
(PPTX)

S6 Fig Effects of the irreversible EGFR-TKI BIBW2992 or the T790M-selective EGFR-TKI WZ4002 on cell growth in H1975, H1975 BIBW-R, and H1975 WZ-R cells. H1975, H1975 BIBW-R, and H1975 WZ-R cells were treated for 72 hours with increasing concentrations of BIBW2992 (left panel) or WZ4002 (right panel). Data generated by cell viability assay (CellTiter-Glo) are expressed as a percentage of the value for untreated cells. The error bars represent SEM of 3 independent experiments.
(PPTX)

S1 Table IC50 values of reagents employed in Fig 4A in HCC4006 and HCC4006ER cells. (DOC)

S2 Table Ranking of the significant pathways in HCC4006ER cells by pathway enrichment analysis based on the results of gene expression microarray. (DOC)

S3 Table Microarray results with fold-change (HCC4006ER:HCC4006) for the genes included in the list of genes negatively correlated with ZEB1 in 38 NSCLC cell lines (See Table 1 and Supplementary Table S2 in ref. [14]). (XLS)

S4 Table Microarray results with fold-change (HCC4006ER:HCC4006) for the genes included in the list of genes positively correlated with ZEB1 in 38 NSCLC cell lines (See Table 2 and Supplementary Table S3 in ref. [14]). (XLS)
