# Supplementary material for: ZEB1 Mediates Acquired Resistance to the Epidermal Growth Factor Receptor-Tyrosine Kinase Inhibitors in Non-Small Cell Lung Cancer
Source: PLoS One. 2016 Jan 20;11(1):e0147344. doi: 10.1371/journal.pone.0147344 (PMC4720447; doi:10.1371/journal.pone.0147344)
Supplement: S1 Fig — HCC4006ER cells cultured in erlotinib-free medium for 2 or 6 months, as well as HCC4006 and the original HCC4006ER cells, were treated for 72 hours with increasing concentrations of erlotinib. Data generated by cell viability assay (CellTiter-Glo) are expressed as a percentage of the value for untreated cells. The error bars represent SEM of 3 independent experiments. (PPTX) [file pone.0147344.s001.pptx]

## Slide 1
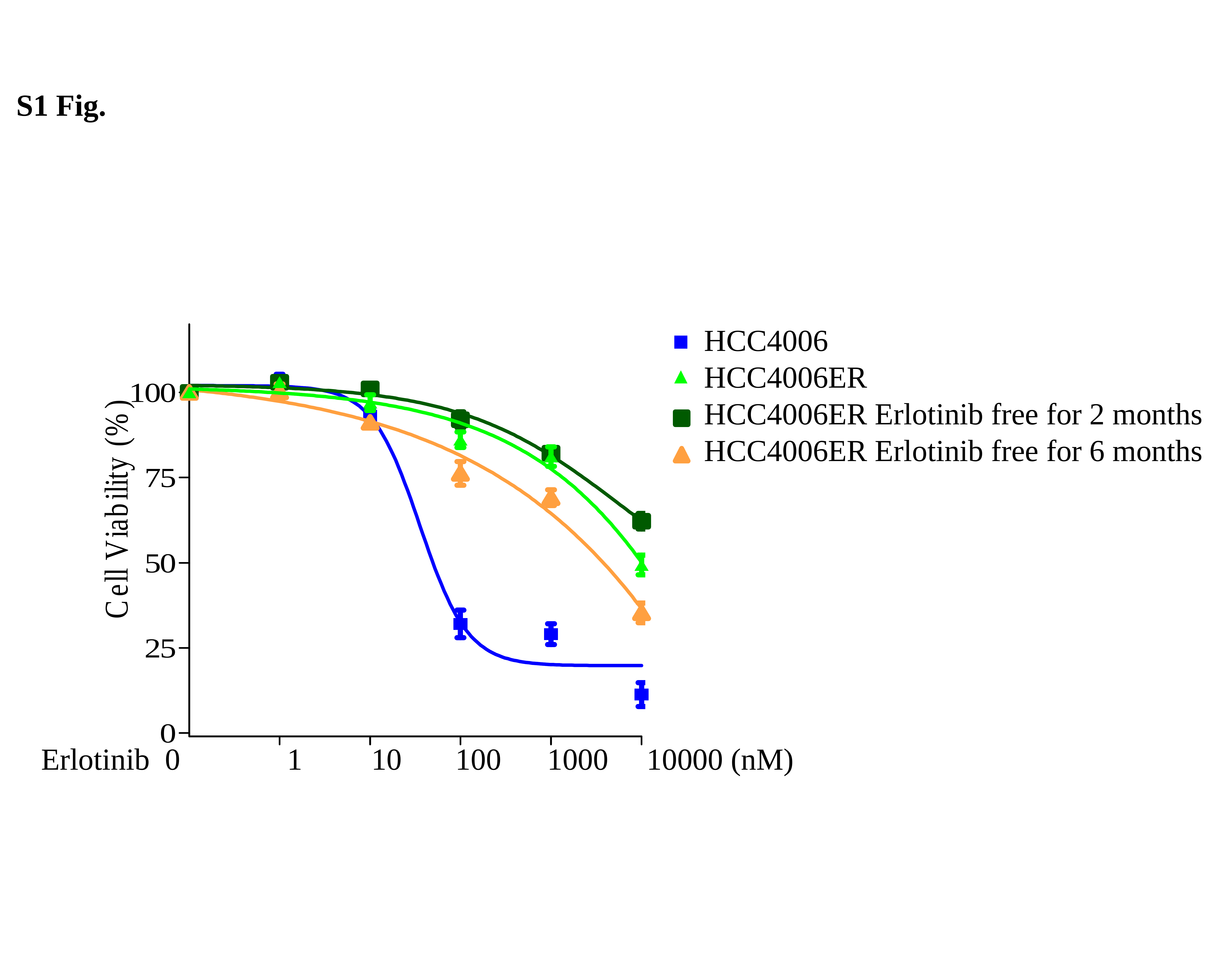

S1 Fig.
HCC4006
HCC4006ER
HCC4006ER Erlotinib free for 2 months
HCC4006ER Erlotinib free for 6 months
Erlotinib 0 　1 10 100 1000 10000 (nM)
