# Supplementary material for: ZEB1 Mediates Acquired Resistance to the Epidermal Growth Factor Receptor-Tyrosine Kinase Inhibitors in Non-Small Cell Lung Cancer
Source: PLoS One. 2016 Jan 20;11(1):e0147344. doi: 10.1371/journal.pone.0147344 (PMC4720447; doi:10.1371/journal.pone.0147344)
Supplement: S2 Fig — A, Single cell clones of HCC4006ER cells (HCC4006ER-S1 to -S5 cells) as well as HCC4006 and the original HCC4006ER cells were treated for 72 hours with increasing concentrations of erlotinib. Data generated by cell viability assay (CellTiter-Glo) are expressed as a percentage of the value for untreated cells. The error bars represent SEM of 3 independent experiments. B, Cell lysates of HCC4006, HCC4006ER, and single cell clones of HCC4006ER cells (HCC4006ER-S1 to -S5 cells) were subjected to protein expression analysis with antibodies to E-cadherin, N-cadherin, vimentin, fibronectin, Her3, and β-actin. (PPTX) [file pone.0147344.s002.pptx]

## Slide 1
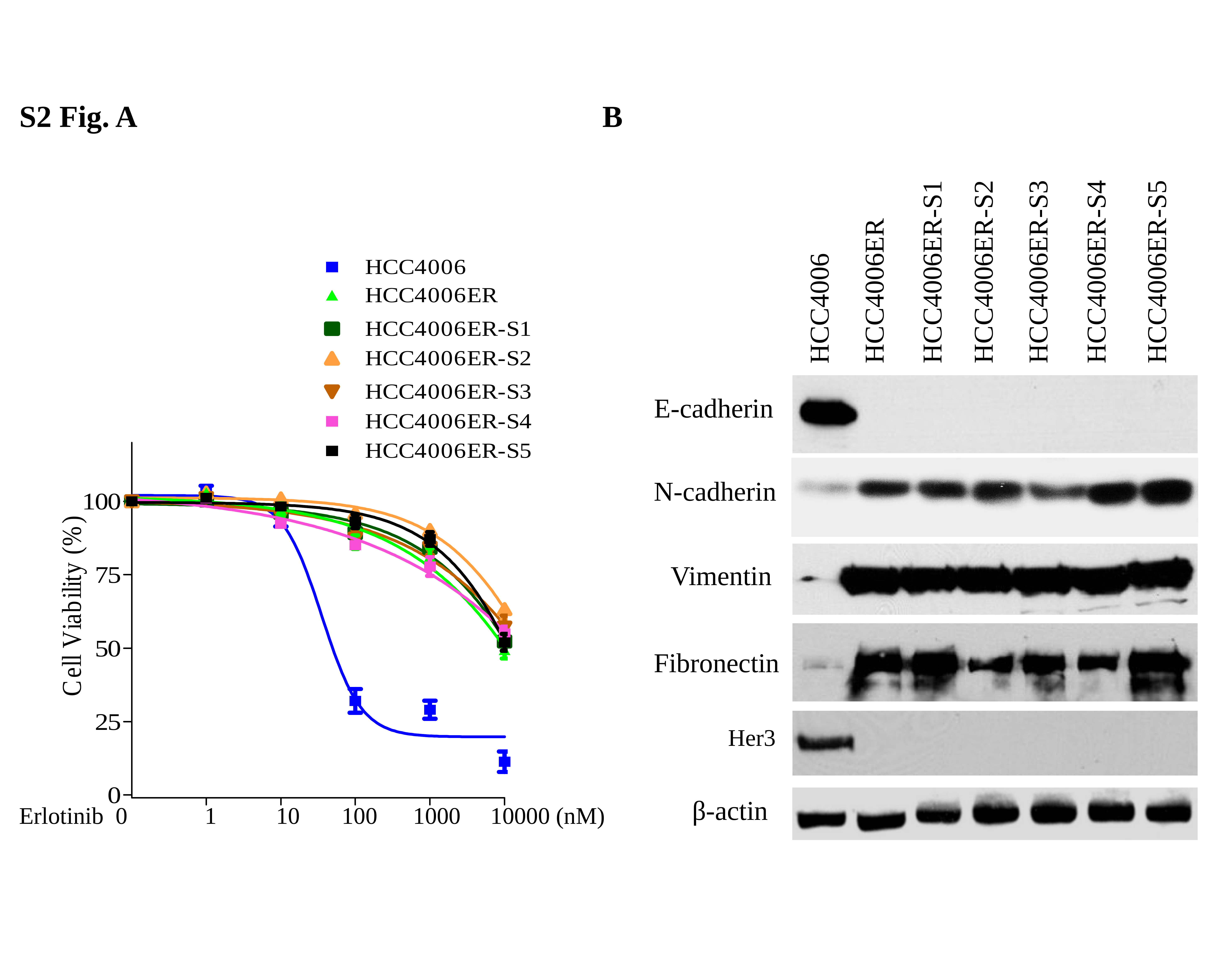

S2 Fig. A B
HCC4006ER-S1
HCC4006ER-S4
HCC4006ER-S2
HCC4006ER-S3
HCC4006ER-S5
HCC4006ER
HCC4006
E-cadherin
N-cadherin
Vimentin
Fibronectin
Her3
β-actin
Erlotinib 0 　1 10 100 1000 10000 (nM)
