# Supplementary material for: ZEB1 Mediates Acquired Resistance to the Epidermal Growth Factor Receptor-Tyrosine Kinase Inhibitors in Non-Small Cell Lung Cancer
Source: PLoS One. 2016 Jan 20;11(1):e0147344. doi: 10.1371/journal.pone.0147344 (PMC4720447; doi:10.1371/journal.pone.0147344)
Supplement: S3 Fig — A, HCC4006 and HCC4006ER cells were incubated for 72 hours ± erlotinib (1 μM). Cell lysates were subjected to protein expression analysis with antibodies to E-cadherin, N-cadherin, vimentin, fibronectin, and β-actin. B, Monolayers of HCC4006 and HCC4006ER cells were scraped in a straight line with a 1000-μL pipette tip. Monolayer photos with scratches were taken after 12-hour incubation with erlotinib (1 μM). (PPTX) [file pone.0147344.s003.pptx]

## Slide 1
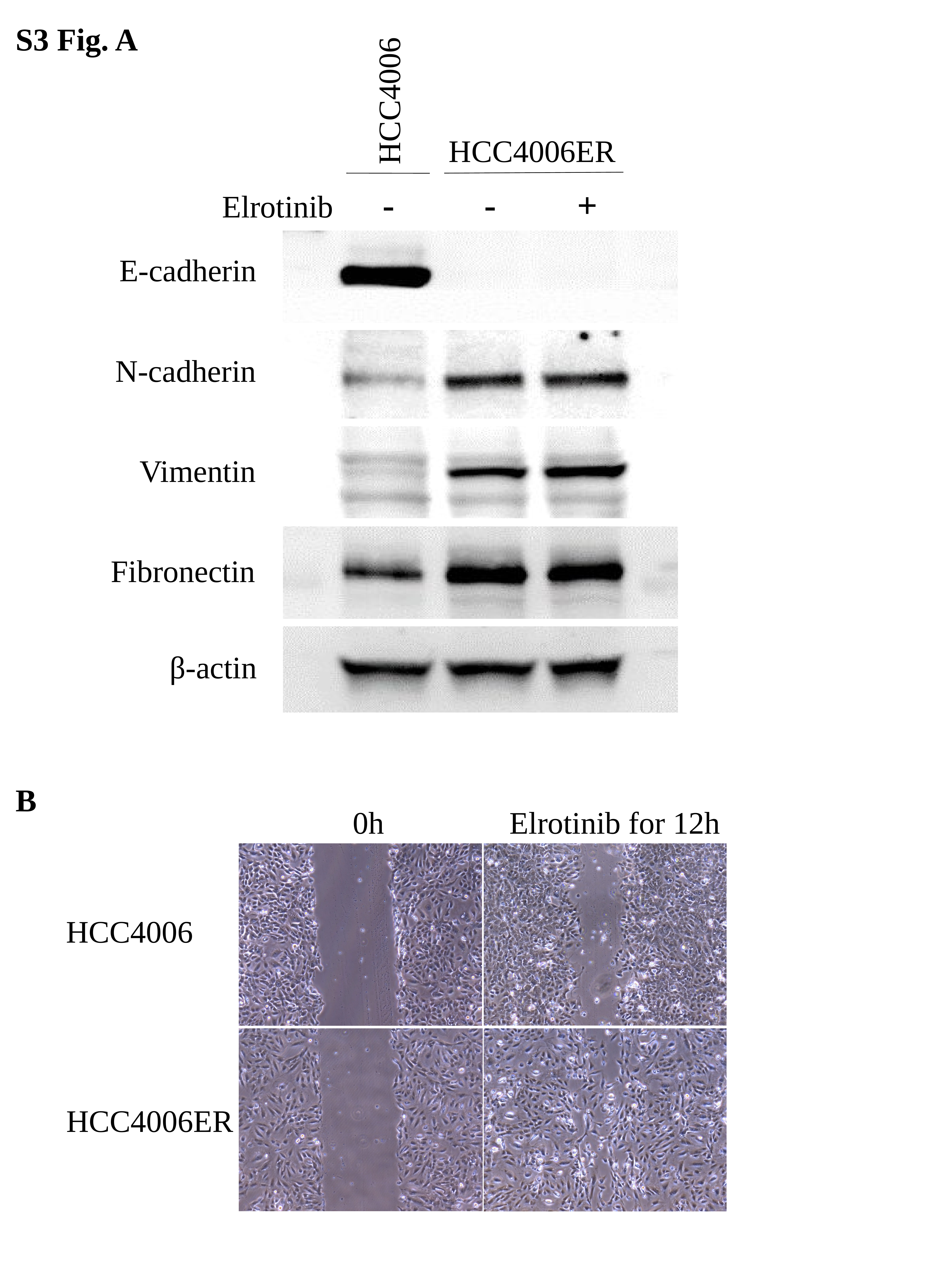

S3 Fig. A
B
HCC4006
HCC4006ER
Elrotinib - - +
E-cadherin
N-cadherin
Vimentin
Fibronectin
β-actin
 0h Elrotinib for 12h
HCC4006
HCC4006ER
