# Supplementary figures and images for: ZEB1 Mediates Acquired Resistance to the Epidermal Growth Factor Receptor-Tyrosine Kinase Inhibitors in Non-Small Cell Lung Cancer
Source: PLoS One. 2016 Jan 20;11(1):e0147344. doi: 10.1371/journal.pone.0147344 (PMC4720447; doi:10.1371/journal.pone.0147344)

## Slide 1
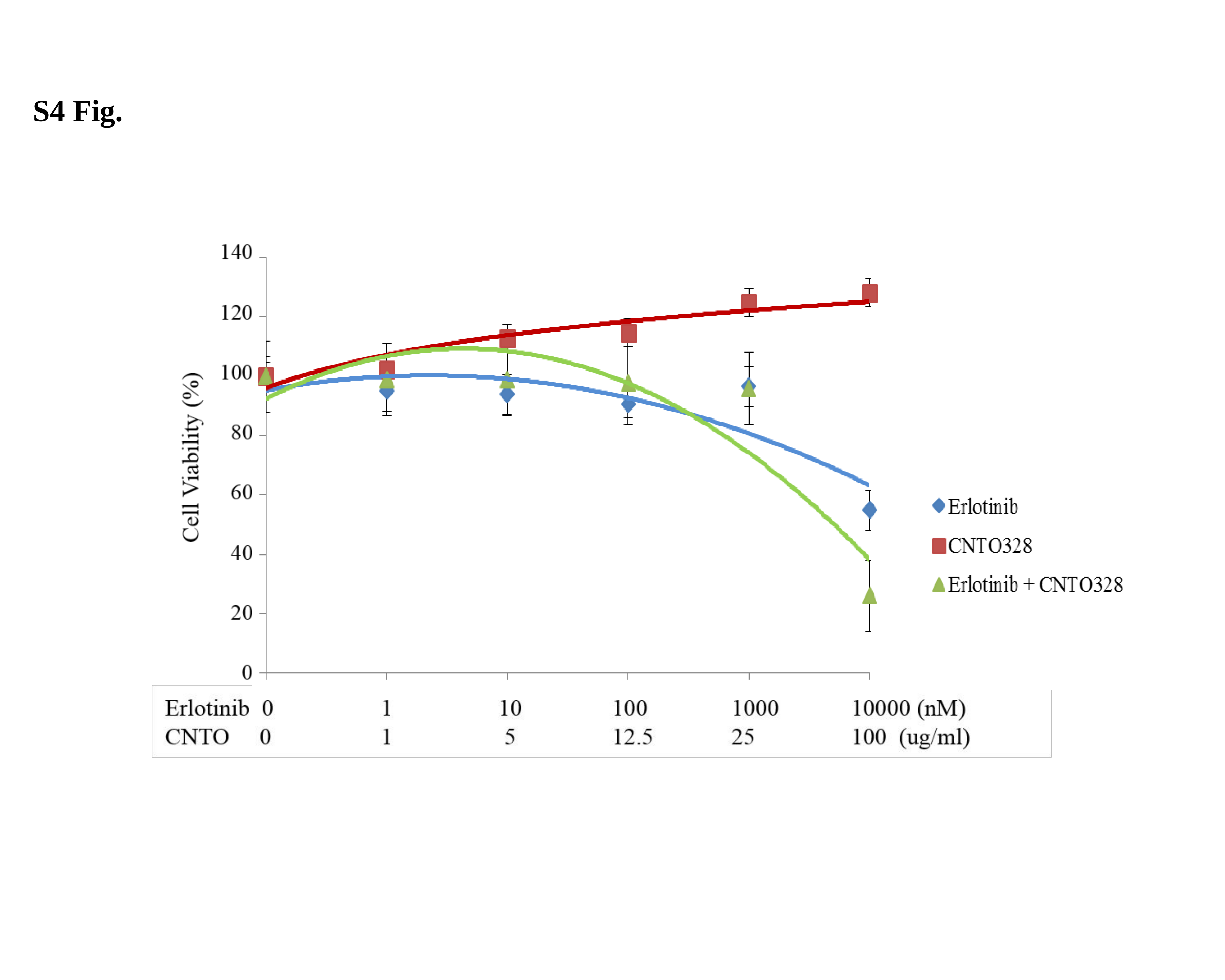

S4 Fig.

Supplement: S4 Fig — HCC4006ER cells were treated for 72 hours with increasing concentrations of erlotinib alone, CNTO328 alone, or erlotinib and CNTO328 in combination. Data generated by cell viability assay (CellTiter-Glo) are expressed as a percentage of the value for untreated cells. The error bars represent SEM of 3 independent experiments. (PPTX) [file pone.0147344.s004.pptx]

## Slide 1
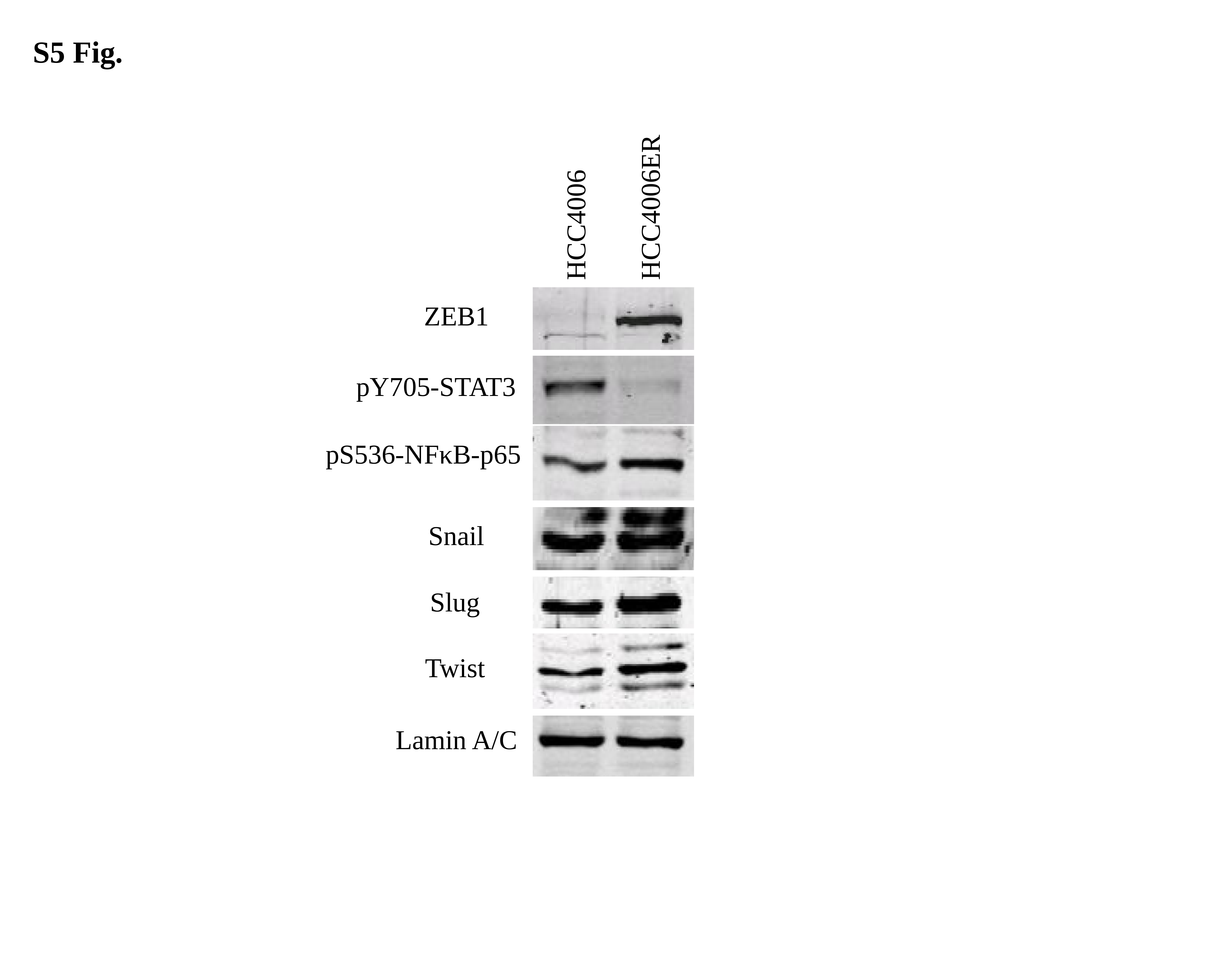

S5 Fig.
HCC4006ER
HCC4006
ZEB1
pY705-STAT3
pS536-NFκB-p65
Snail
Slug
Twist
Lamin A/C

Supplement: S5 Fig — Nuclear extract of both HCC4006 and HCC4006ER cells were subjected to protein expression analysis with antibodies to ZEB1, pT705-STAT3, pS536-NFκB-p65, Snail, Slug, Twist, and Lamin A/C. (PPTX) [file pone.0147344.s005.pptx]

## Slide 1
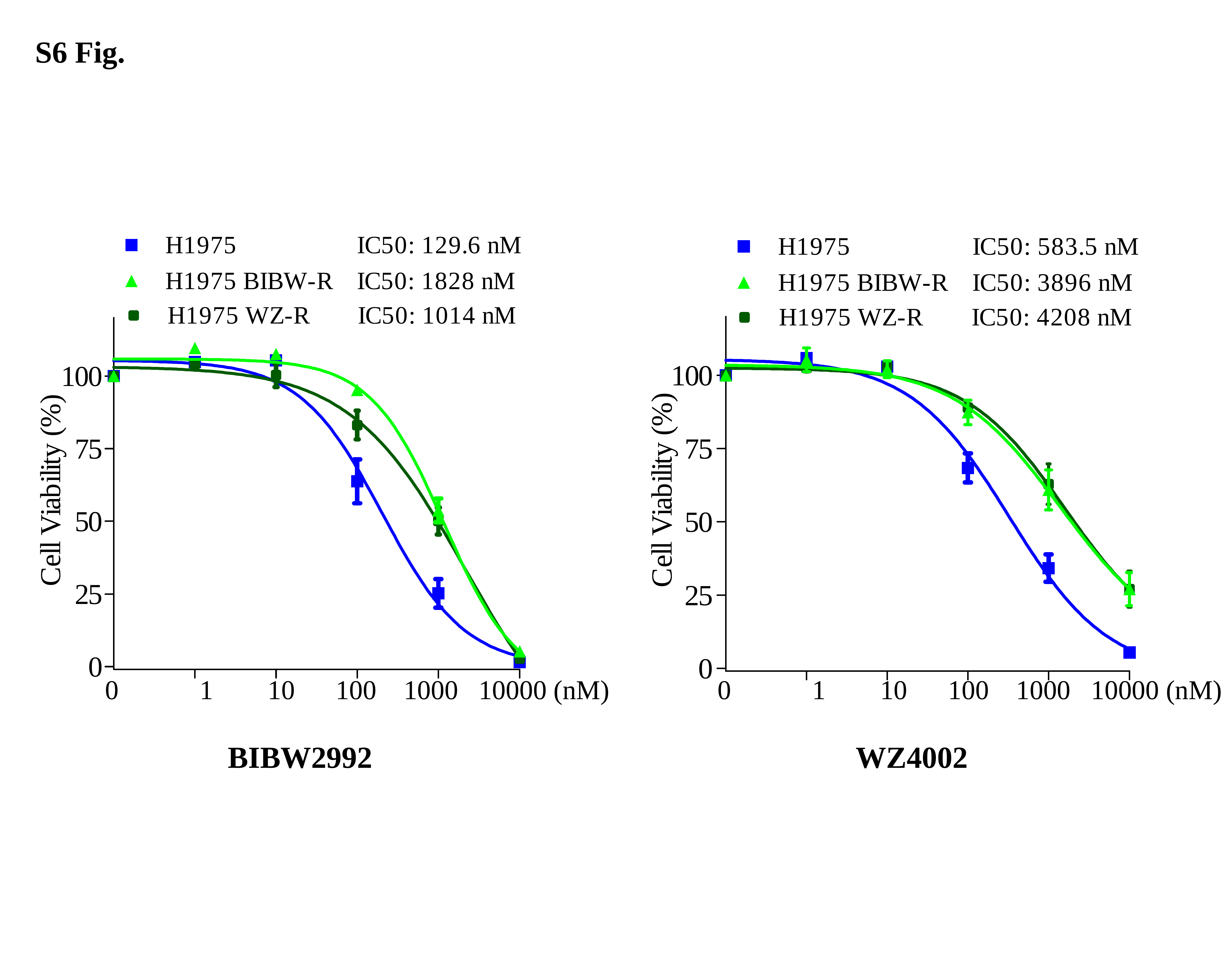

S6 Fig.
0 　1 10 100 1000 10000 (nM)
 BIBW2992
0 　1 10 100 1000 10000 (nM)
 WZ4002

Supplement: S6 Fig — H1975, H1975 BIBW-R, and H1975 WZ-R cells were treated for 72 hours with increasing concentrations of BIBW2992 (left panel) or WZ4002 (right panel). Data generated by cell viability assay (CellTiter-Glo) are expressed as a percentage of the value for untreated cells. The error bars represent SEM of 3 independent experiments. (PPTX) [file pone.0147344.s006.pptx]
