# Supplementary material for: ZEB1 Mediates Acquired Resistance to the Epidermal Growth Factor Receptor-Tyrosine Kinase Inhibitors in Non-Small Cell Lung Cancer
Source: PLoS One. 2016 Jan 20;11(1):e0147344. doi: 10.1371/journal.pone.0147344 (PMC4720447; doi:10.1371/journal.pone.0147344)
Supplement: S1 Table — (DOC) [file pone.0147344.s007.doc]

**S1 Table. IC50 values of reagents employed in Fig 4A in HCC4006 and HCC4006ER cells.**

| **Reagent** | **IC50 (nM)** | |
| --- | --- | --- |
|  | **HCC4006** | **HCC4006ER** |
| PD173074 | >10000 | 9013 |
| LY364947 | >10000 | >10000 |
| LBH589 | 79.8 | 63.4 |
| Salinomycin | 567.2 | 564.7 |
| IWP2 | >10000 | >10000 |
