# Supplementary material for: ZEB1 Mediates Acquired Resistance to the Epidermal Growth Factor Receptor-Tyrosine Kinase Inhibitors in Non-Small Cell Lung Cancer
Source: PLoS One. 2016 Jan 20;11(1):e0147344. doi: 10.1371/journal.pone.0147344 (PMC4720447; doi:10.1371/journal.pone.0147344)
Supplement: S2 Table — (DOC) [file pone.0147344.s008.doc]

**S2 Table. Ranking of the significant pathways in HCC4006ER cells by pathway enrichment analysis based on the results of gene expression microarray.**

| **#** | **Maps** | **pValue** |
| --- | --- | --- |
| 1 | Cytoskeleton remodeling_TGF, WNT and cytoskeletal remodeling | 4.297E-10 |
| 2 | Cell adhesion_Chemokines and adhesion | 9.391E-10 |
| 3 | Cytoskeleton remodeling_Cytoskeleton remodeling | 6.940E-09 |
| 4 | Cell adhesion_Ephrin signaling | 1.511E-08 |
| 5 | Neurophysiological process_Receptor-mediated axon growth repulsion | 1.511E-08 |
| 6 | Development_Regulation of epithelial-to-mesenchymal transition (EMT) | 2.479E-07 |
| 7 | Cell adhesion_ECM remodeling | 3.251E-07 |
| 8 | Cell adhesion_Integrin-mediated cell adhesion and migration | 3.382E-07 |
| 9 | Cytoskeleton remodeling_Reverse signaling by ephrin B | 9.973E-07 |
| 10 | Development_TGF-beta-dependent induction of EMT via SMADs | 1.138E-06 |
